# Genome-Wide Association Study (GWAS) on Bilirubin Concentrations in Subjects with Metabolic Syndrome: Sex-Specific GWAS Analysis and Gene-Diet Interactions in a Mediterranean Population

**DOI:** 10.3390/nu11010090

**Published:** 2019-01-04

**Authors:** Oscar Coltell, Eva M. Asensio, José V. Sorlí, Rocio Barragán, Rebeca Fernández-Carrión, Olga Portolés, Carolina Ortega-Azorín, Raul Martínez-LaCruz, José I. González, Vicente Zanón-Moreno, Ignacio Gimenez-Alba, Montserrat Fitó, Emilio Ros, Jose M. Ordovas, Dolores Corella

**Affiliations:** 1 Department of Computer Languages and Systems, Universitat Jaume I, 12071 Castellón, Spain; oscar.coltell@uji.es; 2 CIBER Fisiopatología de la Obesidad y Nutrición, Instituto de Salud Carlos III, 28029 Madrid, Spain; eva.m.asensio@uv.es (E.M.A.); jose.sorli@uv.es (J.V.S.); rocio.barragan@uv.es (R.B.); Rebeca.Fernandez@uv.es (R.F.-C.); Olga.Portoles@uv.es (O.P.); carolina.ortega@uv.es (C.O.-A.); raulmartinezlacruz@gmail.com (R.M.-L.); Ignacio.Glez-Arraez@uv.es (J.I.G.); Mfito@imim.es (M.F.); EROS@clinic.cat (E.R.); 3 Department of Preventive Medicine and Public Health, School of Medicine, University of Valencia, 46010 Valencia, Spain; nachoga16@gmail.com; 4 Area of Health Sciences, Valencian International University, 46002 Valencia, Spain; vczanon@universidadviu.com; 5 Red Temática de Investigación Cooperativa OftaRed, Instituto de Salud Carlos III, 28029 Madrid, Spain; 6 Ophthalmology Research Unit “Santiago Grisolia”, Dr. Peset University Hospital, 46017 Valencia, Spain; 7 Instituto Hospital del Mar de Investigaciones Médicas, 08003 Barcelona, Spain; 8 Lipid Clinic, Endocrinology and Nutrition Service, Institut d’Investigacions Biomèdiques August Pi Sunyer (IDIBAPS), Hospital Clínic, University of Barcelona, 08036 Barcelona, Spain; 9 Nutrition and Genomics Laboratory, JM-USDA Human Nutrition Research Center on Aging at Tufts University, Boston, MA 02111, USA; jose.ordovas@tufts.edu; 10 Department of Cardiovascular Epidemiology and Population Genetics, Centro Nacional de Investigaciones Cardiovasculares (CNIC), 28029 Madrid, Spain; 11 IMDEA Alimentación, 28049 Madrid, Spain

**Keywords:** bilirubin, GWAS, sex-specific, UGT1A1, gene-diet interaction, Mediterranean

## Abstract

Although, for decades, increased serum bilirubin concentrations were considered a threatening sign of underlying liver disease and had been associated with neonatal jaundice, data from recent years show that bilirubin is a powerful antioxidant and suggest that slightly increased serum bilirubin concentrations are protective against oxidative stress-related diseases, such as cardiovascular diseases. Therefore, a better understanding of the gene-diet interactions in determining serum bilirubin concentrations is needed. None of the previous genome-wide association studies (GWAS) on bilirubin concentrations has been stratified by sex. Therefore, considering the increasing interest in incorporating the gender perspective into nutritional genomics, our main aim was to carry out a GWAS on total serum bilirubin concentrations in a Mediterranean population with metabolic syndrome, stratified by sex. Our secondary aim was to explore, as a pilot study, the presence of gene-diet interactions at the GWAS level. We included 430 participants (188 men and 242 women, aged 55–75 years, and with metabolic syndrome) in the PREDIMED Plus-Valencia study. Global and sex-specific GWAS were undertaken to analyze associations and gene-diet interaction on total serum bilirubin. Adherence (low and high) to the Mediterranean diet (MedDiet) was analyzed as the dietary modulator. In the GWAS, we detected more than 55 SNPs associated with serum bilirubin at *p* < 5 × 10^−8^ (GWAS level). The top-ranked were four SNPs (rs4148325 (*p* = 9.25 × 10^−24^), rs4148324 (*p* = 9.48 × 10^−24^), rs6742078 (*p* = 1.29 × 10^−23^), rs887829 (*p* = 1.39 × 10^−23^), and the rs4148324 (*p* = 9.48 × 10^−24^)) in the UGT1A1 (UDP glucuronosyltransferase family 1 member A1) gene, which replicated previous findings revealing the UGT1A1 as the major locus. In the sex-specific GWAS, the top-ranked SNPs at the GWAS level were similar in men and women (the lead SNP was the rs4148324-UGT1A1 in both men (*p* = 4.77 × 10^−11^) and women (*p* = 2.15 × 10^−14^), which shows homogeneous genetic results for the major locus. There was more sex-specific heterogeneity for other minor genes associated at the suggestive level of GWAS significance (*p* < 1 × 10^−5^). We did not detect any gene-MedDiet interaction at *p* < 1 × 10^−5^ for the major genetic locus, but we detected some gene-MedDiet interactions with other genes at *p* < 1 × 10^−5^, and even at the GWAS level for the IL17B gene (*p* = 3.14 × 10^−8^). These interaction results, however, should be interpreted with caution due to our small sample size. In conclusion, our study provides new data, with a gender perspective, on genes associated with total serum bilirubin concentrations in men and women, and suggests possible additional modulations by adherence to MedDiet.

## 1. Introduction

Genome-wide association studies (GWAS) have allowed the discovery of hundreds of single nucleotide polymorphisms (SNPs) associated with cardiometabolic diseases [1]. However, most of these GWAS were undertaken jointly for men and women, and there is very little data on whether there is any heterogeneity per sex in the gene variants revealed by GWAS [2,3,4,5]. Although several phenotypic sex differences in cardiometabolic diseases have been apparent for decades [6], whether they also exist at the genome level, and consequently influence GWAS results and the subsequent gene-diet interaction studies, has often been overlooked. From the Precision Medicine and Precision Nutrition viewpoints, it is imperative to undertake sex-specific GWAS in order to obtain new information on the most relevant genes and gene variants that may be associated either differentially or homogeneously with cardio-metabolic diseases in men and women [7,8,9,10]. Moreover, it has been suggested that the genetic risk scores (GRS) currently used to predict disease and to test gene-diet interactions, may be skewed and not reflect the correct genetic component, given that the SNPs that have been chosen as the most relevant for constructing the GRS have mostly been obtained from global GWAS, when not taking possible sex differences into account [10]. Thus, it is important to generate new sex-specific GWAS and use the sex-associated SNPs to create sex-specific GRS [10]. Moreover, it is now essential to integrate the gender perspective in any studies undertaken and fill the gap in traditional research when not considering the different characteristics of men and women [11,12,13].

Besides carrying out sex-specific GWAS, it is also crucial in nutritional genomics [14] to generate data on genome-wide sex-specific gene-diet interactions. In traditional GWAS, apart from ignoring the influence of sex, gene-environment interactions were also commonly overlooked [15,16]. Thus, few studies analyzing gene-diet interactions at the GWAS level have been published [17,18,19]. This is, among other factors, due to the large sample size generally required. Moreover, there is a scarcity of studies that have analyzed the heterogeneity/homogeneity by sex in gene-diet interactions at the GWAS level, which is a priority for nutritional genomics.

Therefore, we focused our study on the analysis of sex-specific GWAS and the exploration of gene-diet interactions. We selected the serum concentrations of total bilirubin as the central phenotype taking into account that there is a growing interest in it due to its potential protection against cardiovascular disease [20,21,22,23], and mainly, because it is highly heritable [24]. Hence, statistically significant results at the GWAS level can be obtained with a relatively small number of study subjects. Bilirubin is a tetrapyrrole pigment generated when heme oxygenase catalyzes the degradation of heme. This produces biliverdin, which is converted into bilirubin by biliverdin reductase [25]. Bilirubin is further processed in hepatocytes, where unconjugated bilirubin is conjugated by uridine diphosphate-glucuronosyltransferase (UDP-GT) to a water-soluble form for excretion. In subjects with the Gilbert syndrome, the UDP-GT activity is reduced to 30% of the normal option, which results in hyperbilirubinemia [26]. Several polymorphisms in the UDP-GT family 1 member A1 (UGT1A1) gene, on chromosome 2, have been associated with Gilbert’s syndrome, but the most common one is a TATA box polymorphism (rs8175347), consisting of a (TA)_5–7_ repeat within the UGT1A1 promoter designated UGT1A1*28 [27]. In general, Gilbert’s syndrome is a benign form of unconjugated hyperbilirubinemia [25,26]. However, for other conditions such as liver disease, neonatal jaundice, and kernicterus, it is well known that increased serum bilirubin concentrations are pathological [28,29,30]. Despite its historical association with toxicity, recently bilirubin has been recognized as a powerful antioxidant and anti-inflammatory protective molecule [31,32,33], which is gaining more attention due to its pleiotropic protective effects against several diseases associated with increased oxidative stress [20,31,33]. Thus, for this paradoxical compound, a protective, mainly U-shaped relationship has been reported for the incidence of diabetic retinopathy [34], diabetic kidney disease [35], cardiovascular diseases [20,23,36], cardiovascular and total death [21,37], diabetes [38,39], carotid atherosclerosis [40,41], and kidney disease [42,43] among others [22,44]. Furthermore, recent findings have shown that bilirubin is a novel signaling molecule capable of binding to peroxisome proliferator-activated receptor-alpha PPARα. This direct binding activates the transcriptional activity of PPARα, this is a novel and potentially important function of bilirubin in addition to its antioxidant role. This function may mediate the protection from adiposity provided by moderate increases in bilirubin [45,46].

Despite the well-known sex differences in total serum bilirubin concentrations, which are higher in males than in females [47,48,49], there is still limited information regarding its inverse association with cardiovascular risk in women [20,34,35], and has been suggested that its antioxidant protective effect may be higher in males than in females [48]. Therefore, more studies analyzing bilirubin-related factors by sex are needed. Although some previous GWAS on bilirubin concentrations have been carried out [49,50,51,52,53,54,55,56,57], none has analyzed sex-specific effects, and this information is lacking. Likewise, none of the previous GWAS has investigated gene-diet interactions on bilirubin concentrations. Hence, our aims are: (1) to undertake a GWAS for total serum bilirubin concentrations in a Mediterranean population with a metabolic syndrome to detect the specific genes of that population, including a sex-specific GWAS. (2) To explore (as a pilot analysis) gene-diet interaction at the GWAS level focusing on the Mediterranean diet (MedDiet) pattern, also takes into account the sex-specific differences.

## 2. Materials and Methods

### 2.1. Study Design and Participants

We have carried out a cross-sectional analysis at baseline in participants recruited from the PREDIMED Plus-Valencia study. One of the field centers of the multi-center PREDIMED Plus study, which is an ongoing randomized, primary cardiovascular prevention trial conducted in Spain [58]. A detailed description of the trial is available at http://predimedplus.com/. Baseline data used in this study were obtained from PREDIMED Plus-Valencia participants included in the PREDIMED Plus trial registered at https://doi.org/10.1186/ISRCTN89898870. Eligible participants, recruited from several primary care health facilities in the Valencia field center, were community-dwelling adults (men, 55–75 years, women, 60–75 years) with a body-mass index (BMI) in the overweight or obesity range ((BMI) ≥ 27 and <40 kg/m^2^) and had at least three components of the metabolic syndrome [58]. In the Valencia field center (located on the eastern Mediterranean coast), the total number of randomized participants included in the PREDIMED Plus trial was 465. In this study, we present the analyses of a specific project of our group in the Valencia field center (currently the only center with genome-wide genotyping data). Here we included all the participants in our center with complete data on total baseline bilirubin concentrations and genome-wide genotyping, in addition to other variables for adjustment (*n* = 430). These participants did not differ significantly from our entire sample concerning the main variables. Participants provided written informed consent and study protocols and procedures were approved, according to the ethical standards of the Helsinki Declaration and by the Human Research Ethics Committee of Valencia University, Valencia.

### 2.2. Baseline Anthropometric, Biochemical, and Lifestyle Variables

Anthropometric variables and blood pressure were determined by trained staff and follow the PREDIMED Plus operations protocol [58]. Weight and height were measured with calibrated scales and a wall-mounted stadiometer, respectively. BMI was calculated as the weight in kilograms divided by the height in meters squared. The waist circumference was measured midway between the lowest rib and the iliac crest after normal expiration, using an anthropometric tape. Blood pressure was measured with a validated semi-automatic oscillometer (Omron HEM-705CP, Hoofddorp, The Netherlands) while the participant was in a seated position for 5 min. Blood samples were collected after a 12-h overnight fast. Fasting plasma glucose, total cholesterol, HDL-C, LDL-C, and triglyceride concentrations were measured, as previously described [59]. Standard laboratory analyses were used for the determination of AST and ALT. Serum total bilirubin concentrations were measured using a standardized colorimetric method with the timed-endpoint diazo method with 2,5-dichlorophenyl diazonium.

Type-2 diabetes was defined as previous clinical diagnosis of diabetes, HbA1c levels ≥ 6.5%, or use of anti-diabetic medication [59]. Leisure-time physical activity was assessed using the validated REGICOR questionnaire [60,61], including questions to collect information on the type of activity, frequency (number of days), and duration. The total leisure-time physical activity-related energy expenditure was estimated as the summed product of frequency, duration, and intensity of each activity divided by 30 days/month (MET·min/day). A 17-item screening questionnaire was used for assessing adherence to an energy-restricted Mediterranean diet [62]. Supplemental Table S1 shows the detailed questions included in the 17-item screening questionnaire.

### 2.3. Genome-Wide Genotyping

Genomic DNA was isolated from blood. The quantity of double-stranded DNA was measured using PicoGreen (Invitrogen Corporation, Carlsbad, CA, USA). High-density genotyping was performed at the University of Valencia using the Infinium OmniExpress-24 v1.2 BeadChip genotyping array (Illumina Inc., San Diego, CA, USA), according to the manufacturer’s protocol with appropriate quality standards. This array captures 713,599 markers. Allele detection and genotype calling were performed in the GenomeStudio genotyping module (Illumina, Inc., San Diego, CA, USA). Data cleaning was performed using standard analysis pipelines implemented in the Phyton programing language using the Numpy library modules combined with the PLINK [63,64]. From the initial full set, those SNPs not mapped on autosomal chromosomes were filtered out. In addition, SNPs with a minor allele frequency (MAF) < 0.01 or those that deviated from expected Hardy-Weinberg equilibrium (*p* < 1.0 × 10^−5^) were removed. A total of 622,468 SNPs that passed the quality filter remained for further analysis.

### 2.4. Statistical Analysis

Chi-square tests were used to compare proportions. Student *t*-tests and ANOVA tests were applied to compare crude means of continuous variables. Triglyceride concentrations were log-transformed for the statistical analyses. We analyzed the association using both crude models and adjusted multivariate regression models including potential confounders. When indicated, models were sequentially adjusted as follows: model 1, unadjusted, model 2, adjusted for age and sex, model 3, additionally adjusted for type-2 diabetes, smoking, physical activity, and medications (lipid-lowering drugs, antihypertensive drugs, and insulin) and total adherence to MedDiet (17-item score). For the study of interactions between the SNPs and adherence to MedDiet, we categorized baseline adherence to the MedDiet (obtained by the 17-item screening) into two groups based on the sample median (8 points), defining both groups as “Low” adherence to MedDiet (0–8 points), and “High” adherence to MedDiet (9–17 points). General linear models were used for continuous variables. Adjusted means were estimated for the continuous variables from the corresponding multivariate-adjusted models. Analyses were undertaken for the whole population and stratified by sex. Statistical analyses for descriptive and the selected bilirubin associations (not at the GWAS level) were performed with the IBM SPSS Statistics version 23.0, NY. All tests were two-tailed and *p* values < 0.05 were considered statistically significant for these associations.

For GWAS, genetic association analyses were performed using PLINK v1.9 [63,64]. To evaluate the association of total bilirubin concentrations with each SNP, using PLINK, an additive genetic model was fitted by regressing total bilirubin on genotype dosage (0, 1, or 2 copies of the variant allele). Coefficients for the minor allele were estimated. Unadjusted and adjusted (for sex and age) general linear models were fitted. For the sex-specific GWAS analysis, we carried out the same procedure as for the global GWAS analysis in both men and women strata. In addition to the stratified sex-specific GWAS, we also tested the gene-sex interactions at the GWAS level in the whole population using PLINK GxE tool and showed the statistical significance on the interaction terms gene-sex, as well as the regression coefficients for each stratum. Likewise, the analysis of the gene-diet at the GWAS level was carried out using PLINK and the GxE utility. Adherence to MedDiet as a dichotomous variable was used to test the gene-diet interactions. Stratified analysis of gene-diet interactions by sex was additionally carried out. We used the conventional threshold of *p* < 5 × 10^−8^ (i.e., Bonferroni correction for 1 million tests) for genome-wide statistical significance. Since this threshold is very conservative for a small sample size, SNPs with *p*-values below 1 × 10^−5^ were also considered suggestive of genome-wide significance, and the corresponding results have been shown in some tables and figures for further replication. SNPs were rank-ordered according to the minimum *p*-value in the genetic models.

We used Haploview (Version 4.2) [65] to create Manhattan plots and to calculate LD, and LD-Link to annotate the proxy of the top SNP, respectively. We used RegulomeDB to annotate SNPs with known and predicted regulatory elements in the intergenic regions of the H. sapiens genome [66]. This database allows the annotation of relevant regulatory elements (DNAase hypersensitivity, binding sites of transcription factors, and promoter regions that have been biochemically characterized to regulation transcription), according to several scores. Score 1 indicates higher functionality than score 2, and so on. Scores 4, 5, and 6 indicate little evidence of functionality. Quantile-quantile plot comparing the expected and observed *p*-values [67] was performed in the R-statistical environment.

## 3. Results

**Figure 1:**
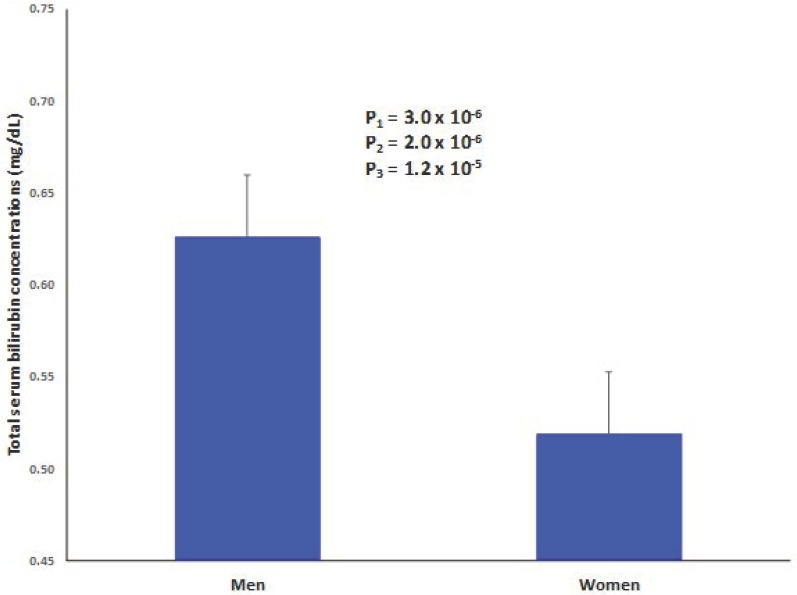
Sex-differences in serum bilirubin concentrations in men and women. Adjusted means for total serum bilirubin concentrations in men (*n* = 188) and women (242). Means adjusted for age, diabetes, body mass index, medications, smoking, physical activity, and adherence to Mediterranean diet (17-item score). The *p*-value for the mean differences between men and women obtained in this multivariate adjusted model is P3. P2: adjusted for age and P1: unadjusted. Error bars: SE of means.

The demographic, lifestyle, and clinical characteristics of the study participants at baseline by sex are presented in Table 1. We analyzed 430 subjects, including 242 women and 188 men, aged 65 ± 5 years, which all harbor the metabolic syndrome. Total serum bilirubin concentrations were higher in men than in women, but, in both groups, the mean value of bilirubin concentrations was within normal limits (≤1 mg/dL generally proposed as reference value) [44]. Figure 1 shows adjusted means for total serum bilirubin concentrations in men and women after adjustment for age, diabetes, BMI, medications, smoking, physical activity, and adherence to the MedDiet. Even after this multivariate adjustment, differences in means by sex still differed significantly (0.63 mg/dL in men versus 0.52 mg/dL in women, *p* = 1.2 × 10^−5^). Even though the mean values of serum bilirubin concentrations were low, 12 participants showed values greater than 1 mg/dL, ranging between 1.1 mg/dL and 1.9 mg/dL, and we excluded these individuals for some interaction analysis.

### 3.1. GWAS Results for Total Serum Bilirubin Concentrations in the Whole Population

Table 2 shows GWAS results for total bilirubin in the whole sample including both men and women. Included in the Table are the 59 top-ranked SNPs with *p*-values for association with bilirubin concentrations at the genome-wide level (*p* < 5 × 10^−8^) of significance in the crude model and/or in the model adjusted for sex and age. The table also shows the regression coefficient, the determination coefficient, the gene name, the minor allele frequency (MAF), and the annotation of relevant regulatory elements, according to RegulomeDB [66]. Scores 1 (a, b, c, d, e, f), 2 (a, b, c), and 3 (a, b) from the RegulomeDB show the predicted functionality of the corresponding SNPs, which are indicated. Scores 4, 5, and 6 indicate little evidence of functionality, which is why they have not been highlighted. As expected, the GWAS carried out in this Mediterranean population revealed the UGT1A gene cluster, located at chromosome 2q37.1, to be the locus most significantly associated with bilirubin concentrations at the GWAS level, which indicates the robustness of our findings. The UGT1A cluster includes nine highly similar protein-coding (UGT1A1, UGT1A3, UGT1A4, UGT1A5, UGT1A6, UGT1A7, UGT1A8, UGT1A9, and UGT1A10) and four non-coding genes, each with a unique alternative first exon followed by a set of common exons 2–5 [68] (Supplemental Figure S1). Each of the UGT1 variable exons is alternatively spliced to the common set of constant exons to produce different mRNA. The top-ranked SNP in our study was the rs4148325-UGT1A1 SNP, with a *p*-value of 9.25 × 10^−24^, after adjustment for sex and age. Similar results were obtained for three other SNPs (rs6742078-UGT1A1, rs887829-UGT1A1, rs4148324-UGT1A1) in the proximal promoter region and intron 1, which is physically close to the well-known UGT1A1*28 TATA box polymorphism (rs8175347). The four top-ranked SNPs (rs4148325-UGT1A1, rs6742078-UGT1A1, rs887829-UGT1A1, and rs4148324-UGT1A). All of these showed similar significant signals with nearly identical regression coefficients (0.149, 0.148, 0.148, and 0.147 mg/dL increase in bilirubin concentrations per variant allele), which were in nearly perfect LD in our study (r^2^ of the rs4148325-UGT1A1 with the s6742078-UGT1A1, rs887829-UGT1A1, and the rs4148324-UGT1A, were: 1, 1, and 0.995, respectively), and in the HapMap CEU population (*r*^2^ = 1.0). These four top-ranked SNPs accounted for approximately 17.4%, 17.3%, 17.1%, and 17.0%, respectively, of the variation in total serum bilirubin levels. Our results for these SNPs were in complete agreement with those reported in the Framingham Heart Study (FHS), the Rotterdam Study (RS), and the Age, Gene, Environment, and Susceptibility-Reykjavik Study (AGES-Reykjavik) [50], which show a remarkable consistency.

**Figure 2:**
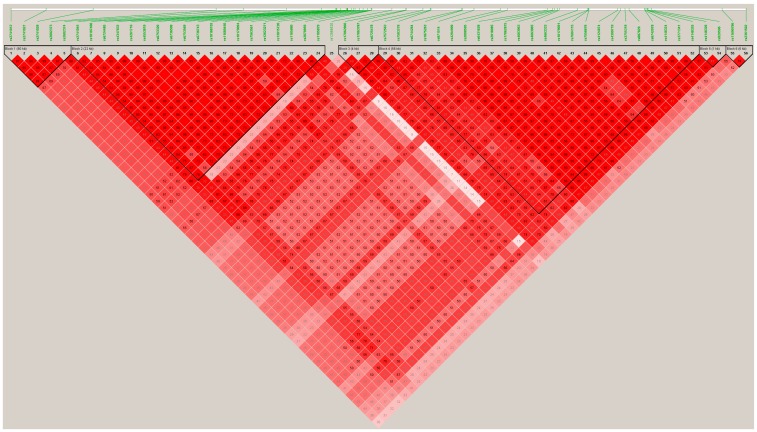
Linkage disequilibrium parameters (*R*^2^) between the top-ranked SNPs in chromosome 2 for the GWAS on total bilirubin concentrations in the 430 participants.

**Figure 3:**
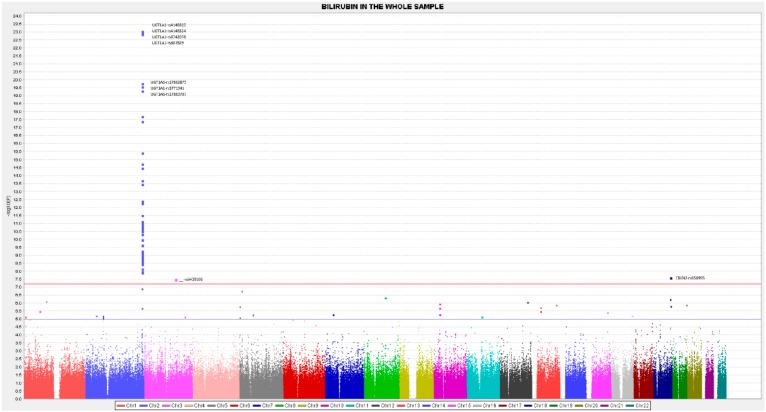
Manhattan plot for the GWAS analysis on total serum bilirubin concentrations in the whole population (*n* = 430 participants), obtained from the genetic additive model adjusted for sex and age, expressed in −log_10_(*p*-value). The blue line represents the threshold 1 (−log_10_(5 × 10^−8^)) for the GWAS statistical significance. The red line represents the threshold 2 ((−log_10_(1 × 10^−5^)).

Apart from the top-ranked SNPs situated in the UGT1A1 gene, we also detected other relevant SNPs at the GWAS level situated in the UGT1A6, UGT1A10, and UGT1A8 genes. Most of these SNPs are found to be in high LD with the lead GWAS SNP rs4148325-UGT1A1. Therefore, they could not be included as independent SNPs in building the GRS for predicting serum bilirubin concentrations, given that the independence criteria for including SNPs in a GRS is considered to have an *r*^2^ < 0.2 with the lead SNP. Supplemental Table S2 presents the LD (*r*^2^) between the lead GWAS SNP rs4148325-UGT1A1 and the other SNPs situated in the cluster of chromosome 2. Figure 2 represents the position of the polymorphisms and their pairwise LD analysis for the entire list of the top-ranked SNPs in the UGT1 cluster. Besides the UGT1A cluster, we have detected a significant association with an intergenic SNP, which is also situated in chromosome 2, the rs2741012 (*p* = 3.22 × 10^−11^, in the sex and age-adjusted model). Figure 3 represents the Manhattan plot of the GWAS for total bilirubin in the whole population, and Supplemental Figure S2, the corresponding Q-Q plot. Additionally, in chromosome 2, we have detected a statistically significant association with two SNPs of the MROH2A (Maestro Heat Like Repeat Family Member 2A) gene, which has previously been associated with high bilirubin concentrations in various GWAS carried out in other populations [50,51,52,53,54]. The percentage of explained variability for this gene (6.8%) is lower than for those of the UGT1A gene. Additionally, at the GWAS-significance level, we have observed an association of bilirubin concentrations with SNPs in the CBLN2 gene (cerebellin 2 precursor). This gene, situated on chromosome 18, has not previously been reported by other GWAS and could represent a more specific association of the characteristics of this Mediterranean population with metabolic syndrome.

### 3.2. Sex-Specific GWAS Results for Total Serum Bilirubin Concentrations in Men and Women

In the stratified GWAS analysis for men and women separately, we have also obtained statistically significant associations at the GWAS level. These statistically significant associations at *p* < 5 × 10^−8^ are presented in Table 3 and Table 4 for men and women, respectively. Bearing in mind that, on reducing the sample size when stratifying for sex, statistical power can be lost and that it would be interesting to know more SNPs that may be associated differentially with bilirubin concentrations in men and women, on the suggestive level of genome-wide significance (*p* < 1 × 10^−5^), Supplemental Tables S3 and S4 present the complete list of top-ranking SNPs for men and women, respectively, from the lead top-ranked SNP to the SNPs with *p* < 1 × 10^−5^. Supplemental Figure S3 shows the Manhattan plots for the sex-specific GWAS in men (A) and women (B). Supplemental Figure S4 shows the corresponding Q-Q plots for the GWAS results in men (A) and women (B). It can be observed that there is an excellent homogeneity in the top-ranked SNPs detected at a GWAS level, both in men and women. In both sexes, the lead GWAS SNP was the rs4148324-UGT1A, which obtained a *p* value, adjusted for age, of 4.77 × 10^−11^ in men and 2.15 × 10^−14^ in women. This SNP forms part of the cluster of the four top-ranked SNPs (rs4148325-UGT1A1, rs6742078-UGT1A1, rs887829-UGT1A1, rs4148324-UGT1A), detected for the whole population. For the following SNPs, the order in the list changes a little depending on significance, but the results are similar for the cluster UGTA1. As the newest result, we should point out that, in women, we detected the SNP rs359935, situated in chromosome 1 (intergenic), as statistically significant at the GWAS level (*p* = 2.17 × 10^−8^), which, as far as we know, is the first time that this has been reported.

Where most heterogeneity exists between men and women is in the SNPs situated in the zone of associations at the suggestive level of genome-wide significance (*p* < 1 × 10^−5^), where we should mention the ASIC2 (acid-sensing ion channel subunit 2), CBLN2, TLL1 (tolloid like 1), ARHGEF38 (Rho Guanine Nucleotide Exchange Factor 38), and SSBP3 (Single-Stranded DNA Binding Protein 3) genes that are detected in men, but not in women. In women, however, we detected associations with the ROM1 (Retinal Outer Segment Membrane Protein 1) and KANK1 (KN Motif and Ankyrin Repeat Domains 1) genes, which do not appear on the list of top-ranked genes in men. These results are reported for the first time and require replication in other studies to confirm their differential significance.

In our study, apart from the sex-specific GWAS, we also analyzed data at the GWAS level including the interactions between sex and each SNP determining serum bilirubin concentrations. Supplemental Table S5 presents the top-ranked SNPs, depending on statistical significance, of the gene-sex interaction term, as well as the values of the regression coefficient on each of the strata (men and women). In line with the homogeneity observed in the stratified analysis, none of the top-ranked SNPs in the cluster of chromosome 2 presents heterogeneity per sex. In general, no statistically significant gene-sex interaction term was obtained at the GWAS level. The first SNP to reach the lower statistical significance of the interaction term (*p* = 1.14 × 10^−7^) is the rs16885705, situated in chromosome 6 (intergenic). The minor allele increases bilirubin concentrations in women, with little effects in men. The next SNP in statistical significance of the interaction term (*p* = 4.8 × 10^−7^) is the rs10484092 in chromosome 14, situated in the FRMD6-AS2 (FRMD6 Antisense RNA 2) gene. Contrary to the previous SNP, the minor allele is associated with higher bilirubin concentrations in men, but not in women.

### 3.3. Gene-Diet Interactions at the GWAS Level in the Whole Population and Sex-Specific Results

As indicated in the Methods section, as the overall diet measurement variable, we analyzed adherence to the MedDiet through a 17-item score. For the analysis of gene-diet interactions, we used the dichotomous variable: high adherence and low adherence. In the gene-diet interaction analyses, we excluded the 12 subjects with serum bilirubin ≥1.1. Table 5 shows the top-ranked SNPs analyzed at the GWAS level and in order of the statistical significance of the gene-MedDiet interaction term in the whole sample.

Only one SNP (rs6887452), located in chromosome 5, reached the statistical significance at this level (*p* = 3.14 × 10^−8^). This SNP is located in the IL17B (Interleukin 17B) gene. The minor allele of this SNP would increase bilirubin concentrations (B = 0.045 mg/dL per minor allele) with low MedDiet adherence while, with high adherence to the MedDiet, the minor allele of this SNPs would be associated with a small decrease of serum bilirubin (B = −0.087 mg/dL). This gene-diet interaction was consistently observed after adjustment for age, diabetes, BMI, smoking, medications, and physical activity. Other SNPs also presented gene-diet interactions in the whole population at *p* < 1 × 10^−5^, including some intergenic variants and variants in the LAMA2 (laminin subunit alpha 2) and EDNRA (Endothelin Receptor Type A) genes. The effect of the minor allele in each dietary strata is detailed in Table 5. There is some heterogeneity on the increasing or decreasing effect of the MedDiet adherence depending on the SNPs analyzed, which suggests the further analysis of diet-specific GRS.

In the sex-specific gene-diet interaction analysis, we detected no SNP reaching the statistical significance at the GWAS level for the interaction term, although one should consider that our sample size is small. However, both for men and women, we detected several SNPs interacting with MedDiet at *p* < 1 × 10^−5^. Supplemental Table S6 shows the top-ranked SNPs for gene-diet interactions in men and Supplemental Table S7 shows the results for women. In this sex-specific analysis, the top-ranked SNPs were heterogeneous for men and women. In men, we detected 3 SNPs, including one in the GRAMD1B (GRAM Domain Containing 1B) gene, which shows opposite effects of the minor allele on serum bilirubin concentrations depending on the adherence to the MedDiet. For women, we detected 16 interacting SNPs at *p* < 1 × 10^−5^ with most of them located in chromosome 10 (intergenic). Other annotated genes were NAT1 (N-Acetyltransferase 1) and PTPRT (Protein Tyrosine Phosphatase, Receptor Type T). Again, the effect of the minor allele of the interacting genes on serum bilirubin concentrations depends on the level of the adherence to the MedDiet, and is heterogeneous depending on the SNP, which adds complexity to the global picture and supports the need for research on diet-specific GRS in addition to the sex-specific analysis.

## 4. Discussion

In this GWAS analysis from a Mediterranean population (composed of European Caucasian subjects) with metabolic syndrome, we confirmed the substantial contribution of the UGT1 gene cluster (chromosome 2) on total serum bilirubin concentrations. Our results at the GWAS level were practically identical to those obtained by other GWAS undertaken in populations of European origin [50,52,54], which shows the high consistency of the results for this major gene. What is more, specifically in the UGT1 gene cluster, the most significant associations at the GWAS level were with 4 SNPs in the UGT1A1. For these SNPs (rs4148325-UGT1A1, rs6742078-UGT1A1, rs887829-UGT1A1, and rs4148324-UGT1A), in nearly perfect LD, located in the promoter region and intron 1, we obtained similar regression coefficients (about 0.148 mg/dL increase in bilirubin concentrations per variant allele) and nearly identical determination coefficients (accounting for 17% of the variation in total serum bilirubin levels), as the values for these parameters reported in the GWAS performed in other European populations, including the FHS, RS, and AGES-Reykjavik studies [50]. Likewise, other GWAS carried out in Chinese [53], Korean [51], or African-American individuals [56], also identified SNPs in the UGT1A1 as the major gene involved in serum bilirubin concentrations. The main differences among populations regarding the major locus are related to the ranking order for the top-ranked SNPs or with their corresponding MAFs. For other minor genes associated with serum bilirubin concentrations, some heterogeneity depending on the population origin that has been reported [50,51,52,53,54]. Thus, the SEMA3C (semaphorin 3C) gene has been detected in GWAS from African-Americans [56] as well as from some European populations [50]. In addition, the associations at the GWAS level with SNPs in other genes of the same cluster, mainly in the UGT1A6 and UGT1A10 genes, are highly significant in our study and, therefore, coincide with previous GWAS [50,51,52,53,54]. However, those studies did not separately analyze men and women. Hence, they do not provide disaggregated data to test the heterogeneity or homogeneity of the genetic influence depending on sex. After many years in which biomedical research studies did not pay attention to the gender perspective [11,13], it is now essential to incorporate the gender perspective or analyze associations in men and women in greater detail in order to uncover the possible similarities or differences [11,12,13]. By carrying out sex-specific GWAS, in our study, we have been able to show that, for the top-ranked SNPs in the major gene determining total serum bilirubin concentrations, there is an excellent homogeneity between men and women. In both sexes, it is the SNPs situated in the UGT1A1 gene, followed by those in the UGT1A6 gene, that are most associated with serum bilirubin concentrations, which emphasizes the great genetic influence of those loci. Nevertheless, although very important, this cluster only explains about 16% to 20% of the variability in bilirubin concentrations. Therefore, other genes in other chromosomes, either with a predominantly genetic influence or through interactions with environmental factors, might contribute to explain another significant percentage of serum bilirubin variability.

In the GWAS analysis for the whole population, we found other SNPs with statistically significant associations at the GWAS level, located in other genes. Outstanding among them is the MROH2A gene that has previously been reported in other GWAS [50,51,52] and which, in our study, explains approximately 6% of the variability of serum bilirubin concentrations. Another gene in our study known as the CBLN2, also presents SNPs associated at the GWAS level. The minor allele of this gene is also associated with higher bilirubin concentrations and would contribute 6% to 7% of the variability. This gene, which may play a role in synaptogenesis induction [69], has been associated with various traits in previous GWAS, including pulmonary arterial hypertension [70] and alcohol consumption [71], but its function is not well known. We did not find any previous GWAS reporting an association with bilirubin concentrations for this SNP. Our association is a particular characteristic of this population (older subjects with metabolic syndrome) or is the result of a false positive. Other GWAS specific for bilirubin have reported associations with other genes such as the SLCO1B1 (solute carrier organic anion transporter family member 1B1) [50,51,52,53]. This function is well known in the metabolism of bilirubin [44]. In our case, we did not detect this gene as being statistically significant at the GWAS level, nor did we find others that functionally are known to participate in the metabolism of bilirubin [44]. This is due to the fact that the contribution in the percentage of explained variability of these genes is lower than those of the UGT1 loci (for example, 1–2% estimated for SLCO1B1) [50,51,52]. Our small sample size does not enable us to detect associations of a lower magnitude than 6% of explained variability as statistically significant at the GWAS level in the whole sample. Thus, to identify new relevant genes, it is necessary to increase the sample size in later studies in Mediterranean populations.

By undertaking the sex-specific GWAS, however, we have been able to show that there is a greater diversity of the genes detected (although our sample size has not allowed us to find associations at the GWAS level, but at the level of suggestive GWAS association (*p* < 1 × 10^−5^)), and that they differ in men and women, which presents a greater heterogeneity that will have to be taken into account if confirmed in later studies. The interest in men is the signal obtained with SNP rs11942650 situated in the TLL1 gene with *p* = 4.27 × 10^−6^, after adjustment for age. This gene has recently been associated with a higher risk of developing hepatocellular carcinoma, probably via hepatic fibrogenesis [72], and its association with that phenotype in patients still in the early stages of the disease could be responsible for the statistically significant signal that we have found in the higher bilirubin concentrations of men in our study. This gene has not been found in women in the corresponding analysis. In contrast, in women, the genes found and associated with serum bilirubin at *p* < 1 × 10^−5^ were ROM1 and KANK1. The ROM1 gene is a member of a photoreceptor-specific gene family and variations in this gene have been associated with retinitis pigmentosa [73]. The possible link between this disease and bilirubin concentrations is unknown. However, for the other gene, KANK1, there may be a stronger relationship, given that deletions in this gene have been associated with cerebral palsy and alterations in neurodevelopment [74] and it is known that, in the case of extreme hyperbilirubinemia in the newborn neurological sequelae can occur, including the Kernicterus Spectrum Disorders [30,75]. However, our data on this level of statistical significance only provide suggestive results for later replication.

Interest in the study of the factors associated with serum bilirubin concentrations has increased recently since there is more evidence of its antioxidant and anti-inflammatory effects [22,31,32,33] and its protective effect (U-shaped) against the incidence of diabetes, kidney disease, cardiovascular disease, other cardiovascular phenotypes, and even mortality [20,21,23,34,35,36,37,38,39,40,41,42,43,44]. Initially, the focus was between the association of high bilirubin concentrations and jaundice in children and other related syndromes (Gilbert, etc.) as well as liver damage in adults [28,29,30,75]. However, even in Gilbert’s syndrome [26], associated with SNPs in the UGT1A1 gene and a mild hyperbilirubinemia, a lower incidence of cardiovascular diseases has been reported [76]. An explanation for this protection may be bilirubin’s ability to inhibit multiple processes that induce platelet hyper-reactivity and thrombosis, which have not been widely recognized until recently [22,31]. Moreover, the protective role of bilirubin has been reported even for subjects with a high range but not exceeding the normal range of values for serum bilirubin concentrations [20,22]. These values are lower than those present in Gilbert’s syndrome [26]. For all those reasons, as well as investigating the central genes involved in the genetic influence, it is also necessary to study the environmental factors that modulate bilirubin concentrations. Several years ago, the “Iatrogenic Gilbert syndrome” was proposed as a strategy for reducing vascular risk by increasing plasma-unconjugated bilirubin by specific medications [77]. Currently, the dietary modulation could provide an alternative approach. Previous studies that analyzed the relationship between diet and serum bilirubin concentrations were mainly aimed at investigating the components of the diet that could contribute to reducing bilirubin concentrations in situations where they were pathologically high [78,79,80].

Nevertheless, current knowledge suggests that, within the normal range, slightly higher serum bilirubin concentrations may have an anti-oxidizing protective effect. Thus, it would be interesting to know whether such concentrations could be slightly increased through dietary intervention, especially in individuals with gene variants associated with genetically lower concentrations. In our study, we have focused on analyzing gene-diet interactions, concentrating on adherence to the MedDiet as the primary variable, and looking at both increasing and decreasing effects depending on the SNP and the level of adherence to the MedDiet. Despite the limitations of a small sample size, we have been able to show that, for the main SNPs in the UGT1A1 cluster, there was no statistically significant gene-MedDiet interaction, neither in the whole population or for either men or women, which suggests that it might be difficult to modulate the significant genetic effect of main variants through diet, or at least with adherence to the MedDiet as assessed here. This is necessary to study other more specific dietary components of diet to detect additional interactions with the major bilirubin-related genes. For other gene variants that do not have such a strong genetic contribution in the association GWAS, we did find some statistically significant gene-diet interactions at *p* < 5 × 10^−5^ both for the whole sample and for men and women. For the whole sample, we have obtained a gene-MedDiet interaction on serum bilirubin concentrations at the GWAS level involving the rs6887452 SNP (an upstream variant) in the IL17B gene. The interleukin-17 family of cytokines has emerged as a critical player in inflammatory diseases with the IL-17B being an anti-inflammatory cytokine [81]. According to our results, the minor allele of the rs6887452-IL17B SNP is associated with higher or lower serum bilirubin concentrations depending on adherence to the MedDiet. This is the first time that this and the other sex-specific gene-diet interactions have been reported at the suggestive level of GWAS significance, and we do not fully understand the mechanisms involved. Some could be false positives, and future studies are required to characterize them. Our results provide new data suggesting that, to generate evidence on gene-diet interactions that can be applied to the new precision nutrition [14], sex-specific studies have to be undertaken to characterize whether there are important sex/gender differences because not all the gene variants involved will be homogeneous or heterogeneous per sex.

## 5. Conclusions

In conclusion, our results in the general GWAS for bilirubin concentrations in subjects with metabolic syndrome from a Mediterranean population are robust and support the great importance of various SNPs in the UGT1A1gene associated with the total serum bilirubin concentrations reported in other studies. These associations with the top-ranked SNPs were homogeneous in men and women in the sex-specific GWAS. However, for other SNPs with a minor genetic contribution, there was more diversity between men and women. The study of gene-diet interactions at the GWAS level, considering adherence to MedDiet, has allowed us to observe some gene-diet interactions at the suggestive level of GWAS significance, both in the whole sample and in men and women. Therefore, this indicates the need for further studies that incorporate the gender perspective in order to obtain more useful results in precision nutrition. Our novel gene-diet interaction results require replication and a more in-depth study in other populations.

## Figures and Tables

**Table 1:** Demographic, clinical, lifestyle, and genetic characteristics of the study participants at baseline according to sex.

	Total (*n* = 430)	Men (*n* = 188)	Women (*n* = 242)	*p*
Age (years)	65.1 ± 0.2	63.9 ± 0.4	66.1 ± 0.3	<0.001
Weight (Kg)	84.5 ± 0.7	92.8 ± 1.0	78.0 ± 0.6	<0.001
BMI (Kg/m^2^)	32.4 ± 0.2	32.3 ± 0.2	32.5 ± 0.2	0.629
Waist circumference (cm)	106.1 ± 0.5	111.2 ± 0.6	102.0 ± 0.6	<0.001
SBP (mm Hg)	141.6 ± 0.9	143.8 ± 1.3	139.9 ± 1.2	0.026
DBP (mm Hg)	81.0 ± 0.5	82.6 ± 0.7	79.7 ± 0.6	0.002
Total cholesterol (mg/dL)	196.4 ± 1.8	188.3 ± 2.8	202.6 ± 2.3	<0.001
LDL-C (mg/dL)	125.0 ± 1.5	121.6 ± 2.4	127.7 ± 1.9	0.044
HDL-C (mg/dL)	51.5 ± 0.5	47.5 ± 0.8	54.7 ± 0.7	<0.001
Triglycerides (mg/dL)	141.6 ± 2.9	138.2 ± 3.8	144.3 ± 4.2	0.296
Fasting glucose (mg/dL)	112.5 ± 1.3	112.8 ± 2.0	112.3 ± 1.7	0.862
Bilirubin (mg/dL)	0.58 ± 0.01	0.64 ± 0.02	0.53 ± 0.01	<0.001
AST (U/L)	26.4 ± 0.4	27.7 ± 0.7	25.4 ± 0.5	0.005
ALT (U/L)	28.4 ± 0.8	30.8 ± 1.2	26.6 ± 1.0	0.008
Type 2 diabetes: *n*, %	169 (39.3)	74 (39.4)	95 (39.3)	0.982
Current smokers: *n*, %	49 (11.4)	30 (16.0)	19 (7.9)	<0.001
Medications: *n*, %				
Antihypertensive drugs	339 (78.8)	148 (78.7)	191 (78.9)	0.959
Hypolipidemic drugs	278 (64.7)	125 (66.5)	153 (63.2)	0.482
Insulin	22 (5.1)	11 (5.9)	11 (4.5)	0.542
Physical Activity (MET.min/week)	1715 ± 77	1940 ± 134	1539 ± 89	0.227
Adherence to MedDiet (P17) ^1^	7.97 ± 0.13	7.79 ± 0.20	8.12 ± 0.18	0.010

Values are mean ± SE for continuous variables and number (%) for categorical variables. BMI indicates body mass index. MedDiet, Mediterranean diet. *p*: *p*-value for the comparisons (means or %) between male and female individuals. AST: Aspartate transaminase. ALT: Alanine transaminase. MET: Metabolic Equivalent. 1 MET is equivalent to kcal·kg^−1^·h^−1^, the oxygen cost of sitting quietly measured as 3.5 mL/kg/min. ^1^: Quantitative 17-item questionnaire for Adherence to Mediterranean diet (MedDiet). SBP: Systolic Blood Pressure. DBP: Diastolic Blood Pressure.

**Table 2:** GWAS results (the top-ranked SNPs) for the association between bilirubin concentrations in the whole sample.

CHR	SNP	BETA	SE	*R* ^2^	*p* ^1^	*p* ^2^	MA	MAF	Gene
2	rs4148325	0.149	0.016	0.174	1.97 × 10^−19^	9.25 × 10^−24^	C	0.354	UGT1A1
2	rs6742078	0.148	0.016	0.173	2.84 × 10^−19^	1.29 × 10^−23^	T	0.348	UGT1A1
2	rs887829	0.148	0.016	0.171	3.72 × 10^−19^	1.39 × 10^−23^	T	0.354	UGT1A1
2	rs4148324	0.147	0.016	0.170	4.62 × 10^−19^	9.48 × 10^−24^	G	0.353	UGT1A1
2	rs17863787	0.142	0.016	0.157	1.40 × 10^−17^	4.70 × 10^−20^	G	0.263	UGT1A6 ^4^
2	rs17862875	0.146	0.017	0.152	4.46 × 10^−17^	1.68 × 10^−20^	A	0.295	UGT1A6
2	rs3771341	0.143	0.017	0.150	8.64 × 10^−17^	2.65 × 10^−20^	A	0.330	UGT1A1
2	rs6744284	0.141	0.017	0.144	4.39 × 10^−16^	4.06 × 10^−18^	T	0.390	UGT1A6
2	rs929596	0.136	0.017	0.132	8.14 × 10^−15^	1.83 × 10^−18^	G	0.324	UGT1A1
2	rs2070959	0.127	0.016	0.127	2.47 × 10^−14^	3.78 × 10^−16^	G	0.278	UGT1A6
2	rs1105879	0.121	0.016	0.123	8.14 × 10^−14^	1.84 × 10^−15^	C	0.325	UGT1A6
2	rs1105880	0.120	0.016	0.120	1.55 × 10^−13^	3.30 × 10^−15^	G	0.343	UGT1A6
2	rs2741045	0.119	0.017	0.109	2.50 × 10^−12^	2.03 × 10^−14^	T	0.159	UGT1A10
2	rs7583278	0.111	0.016	0.105	7.21 × 10^−12^	3.87 × 10^−13^	T	0.391	UGT1A6
2	rs2018985	0.111	0.016	0.103	1.19 × 10^−11^	3.49 × 10^−14^	G	0.441	UGT1A6
2	rs6725478	0.110	0.016	0.102	1.22 × 10^−11^	5.42 × 10^−13^	T	0.388	UGT1A6
2	rs7604115	0.106	0.016	0.088	3.28 × 10^−10^	3.09 × 10^−12^	T	0.350	UGT1A6
2	rs3755319	0.102	0.016	0.086	7.53 × 10^−10^	7.28 × 10^−12^	A	0.450	UGT1A1
2	rs4148326	0.101	0.016	0.085	8.03 × 10^−10^	8.20 × 10^−12^	T	0.480	UGT1A1
2	rs4663965	0.101	0.016	0.084	1.00 × 10^−09^	1.10 × 10^−11^	T	0.415	UGT1A6
2	rs4124874	0.100	0.016	0.083	1.04 × 10^−09^	9.01 × 10^−12^	T	0.412	UGT1A6 ^3^
2	rs2008595	0.101	0.016	0.083	1.08 × 10^−09^	1.67 × 10^−11^	C	0.412	UGT1A6
2	rs1875263	0.102	0.016	0.084	1.10 × 10^−09^	1.02 × 10^−10^	T	0.417	UGT1A6 ^3^
2	rs6431628	0.100	0.016	0.083	1.29 × 10^−09^	1.30 × 10^−11^	A	0.410	UGT1A6
2	rs2741027	0.102	0.016	0.083	1.32 × 10^−09^	9.63 × 10^−12^	A	0.177	__
2	rs10179091	0.100	0.016	0.083	1.34 × 10^−09^	1.01 × 10^−11^	T	0.482	UGT1A6
2	rs4663333	0.100	0.016	0.083	1.35 × 10^−09^	1.38 × 10^−11^	G	0.411	UGT1A6
2	rs4663963	0.100	0.016	0.083	1.35 × 10^−09^	1.35 × 10^−11^	T	0.420	UGT1A6
2	rs871514	0.100	0.016	0.082	1.37 × 10^−09^	1.82 × 10^−11^	T	0.428	UGT1A6
2	rs10197460	0.099	0.016	0.083	1.42 × 10^−09^	2.32 × 10^−10^	T	0.298	UGT1A10
2	rs2741029	0.102	0.017	0.081	1.79 × 10^−09^	1.07 × 10^−11^	G	0.177	UGT1A8
2	rs4399719	0.099	0.016	0.081	1.89 × 10^−09^	1.63 × 10^−11^	T	0.415	UGT1A6
2	rs2602373	0.102	0.017	0.080	2.16 × 10^−09^	1.51 × 10^−11^	C	0.177	UGT1A10
2	rs2602374	0.105	0.017	0.080	2.16 × 10^−09^	2.36 × 10^−11^	T	0.173	UGT1A10
2	rs4294999	0.098	0.016	0.079	3.15 × 10^−09^	4.78 × 10^−11^	A	0.426	UGT1A6
18	rs658995	0.286	0.047	0.079	3.33 × 10^−09^	2.50 × 10^−08^	A	0.282	CBLN2
2	rs10179094	0.096	0.016	0.078	3.75 × 10^−09^	5.34 × 10^−10^	A	0.297	UGT1A10 ^3^
2	rs2741012	0.098	0.016	0.078	4.11 × 10^−09^	3.22 × 10^−11^	T	0.191	__
2	rs6724485	0.095	0.016	0.077	4.50 × 10^−09^	1.12 × 10^−09^	A	0.358	UGT1A10
2	rs12988520	−0.098	0.016	0.077	5.01 × 10^−09^	6.94 × 10^−10^	C	0.470	UGT1A6
2	rs13015720	0.096	0.016	0.077	5.06 × 10^−09^	9.86 × 10^−10^	A	0.362	UGT1A6
2	rs7572563	−0.098	0.016	0.077	5.43 × 10^−09^	1.03 × 10^−09^	G	0.403	UGT1A6
2	rs4261716	0.095	0.016	0.076	6.40 × 10^−09^	1.45 × 10^−09^	T	0.358	UGT1A10
2	rs17862866	−0.097	0.016	0.076	6.86 × 10^−09^	1.12 × 10^−09^	A	0.402	UGT1A6
2	rs6736743	0.094	0.016	0.075	7.24 × 10^−09^	1.69 × 10^−09^	A	0.362	UGT1A10
2	rs6736508	0.094	0.016	0.075	7.25 × 10^−09^	1.68 × 10^−09^	A	0.362	UGT1A10
2	rs12623271	0.094	0.016	0.075	7.65 × 10^−09^	1.75 × 10^−09^	G	0.362	UGT1A6
2	rs10168155	0.094	0.016	0.075	7.87 × 10^−09^	1.69 × 10^−09^	T	0.362	UGT1A10
2	rs6753320	0.093	0.016	0.074	1.03 × 10^−08^	2.30 × 10^−09^	C	0.363	UGT1A10
2	rs7563561	0.093	0.016	0.074	1.03 × 10^−08^	2.30 × 10^−09^	G	0.362	UGT1A10
2	rs4553819	0.093	0.016	0.074	1.05 × 10^−08^	2.43 × 10^−09^	G	0.358	UGT1A10
2	rs6753569	0.093	0.016	0.074	1.09 × 10^−08^	2.39 × 10^−09^	C	0.362	UGT1A10
2	rs4347832	0.093	0.016	0.074	1.13 × 10^−08^	2.52 × 10^−09^	C	0.358	UGT1A10
2	rs11680450	0.092	0.016	0.072	1.73 × 10^−08^	3.54 × 10^−09^	C	0.362	UGT1A10
2	rs7556676	0.093	0.017	0.069	3.51 × 10^−08^	6.99 × 10^−10^	G	0.497	UGT1A6
2	rs2361502	0.096	0.017	0.069	3.69 × 10^−08^	1.17 × 10^−08^	C	0.282	MROH2A
2	rs11690786	0.096	0.017	0.067	6.38 × 10^−08^	7.05 × 10^−09^	T	0.281	MROH2A
18	rs595333	0.252	0.046	0.066	7.46 × 10^−08^	5.81 × 10^−07^	T	0.291	CBLN2
3	rs6439106	0.409	0.077	0.062	1.79 × 10^−07^	3.14 × 10^−08^	T	0.221	__

CHR: Chromosome. SNP: Single Nucleotide Polymorphism. Beta: indicates the regression coefficients showing the increase or decrease in the bilirubin concentrations (mg/dL) per minor allele. SE: Standard error of Beta. *R*^2^: Indicates the determination coefficient (variability explained). MA: Minor allele. MAF: minor allele (MA) frequency. *n* = 430 subjects analyzed. ^1^: Unadjusted *p*-value in the additive model. ^2^: adjusted *p*-value for sex and age (additive model). ^3^: SNP scored as 2b in the RegulomeDB with the meaning of likely to affect binding. ^4^: SNP scored as 3a in the RegulomeDB with the meaning being less likely to affect binding.

**Table 3:** GWAS results for the association between bilirubin concentrations and the top-ranked SNPs (at the GWAS level: *p* < 5 × 10^−8^) in men.

CHR	SNP	Beta	SE	*R* ^2^	*p* ^1^	*p* ^2^	MA	MAF	Gene
2	rs4148324	0.177	0.027	0.189	4.44 × 10^−10^	4.77 × 10^−11^	G	0.353	UGT1A1
2	rs887829	0.176	0.027	0.188	5.89 × 10^−10^	7.04 × 10^−11^	T	0.354	UGT1A1
2	rs3771341	0.174	0.028	0.166	6.20 × 10^−09^	1.20 × 10^−09^	A	0.330	UGT1A1
2	rs17862875	0.173	0.029	0.161	1.00 × 10^−08^	2.40 × 10^−09^	A	0.295	UGT1A6
2	rs929596	0.171	0.029	0.154	2.29 × 10^−08^	3.75 × 10^−09^	G	0.324	UGT1A1
2	rs17863787	0.159	0.028	0.149	4.37 × 10^−08^	1.04 × 10^−08^	G	0.263	UGT1A6 ^3^
2	rs1105879	0.150	0.027	0.144	8.14 × 10^−08^	1.07 × 10^−08^	C	0.325	UGT1A6
2	rs2070959	0.155	0.028	0.143	8.44 × 10^−08^	2.15 × 10^−08^	G	0.278	UGT1A6

CHR: Chromosome. SNP: Single Nucleotide Polymorphism. Beta: indicates the regression coefficients showing the increase or decrease in the bilirubin concentrations (mg/dL) per minor allele. SE: Standard error of Beta. *R*^2^: Indicates the determination coefficient (variability explained). MA: Minor allele. MAF: minor allele (MA) frequency. *n* = 188 subjects analyzed. ^1^: Unadjusted *p*-value in the additive model. ^2^: Adjusted *p*-value for age (additive model). ^3^: SNP scored as 3a in the RegulomeDB with the meaning of less likely to affect binding.

**Table 4:** GWAS results for the association between bilirubin concentrations and the top-ranked SNPs (at the GWAS level: *p* < 5 × 10^−8^) in women.

CHR	SNP	Beta	SE	*R* ^2^	*p* ^1^	*p* ^2^	MA	MAF	Gene
2	rs4148324	0.142	0.017	0.218	1.99 × 10^−14^	2.15 × 10^−14^	G	0.353	UGT1A1
2	rs6742078	0.142	0.018	0.217	2.69 × 10^−14^	2.97 × 10^−14^	T	0.348	UGT1A1
2	rs17863787	0.135	0.017	0.202	2.14 × 10^−13^	2.40 × 10^−13^	G	0.263	UGT1A6 ^3^
2	rs17862875	0.141	0.018	0.199	3.61 × 10^−13^	4.03 × 10^−13^	A	0.295	UGT1A6
2	rs3771341	0.136	0.018	0.189	1.52 × 10^−12^	1.67 × 10^−12^	A	0.330	UGT1A1
2	rs6744284	0.134	0.018	0.182	5.16 × 10^−12^	5.70 × 10^−12^	T	0.390	UGT1A6
2	rs929596	0.128	0.019	0.166	4.48 × 10^−11^	4.65 × 10^−11^	G	0.324	UGT1A1
2	rs2018985	0.115	0.018	0.147	9.23 × 10^−10^	1.00 × 10^−09^	G	0.441	UGT1A6
2	rs2070959	0.111	0.018	0.139	2.40 × 10^−09^	2.57 × 10^−09^	G	0.278	UGT1A6
2	rs2741045	0.108	0.018	0.128	1.08 × 10^−08^	1.07 × 10^−08^	T	0.159	UGT1A10
1	rs359935	0.353	0.061	0.126	1.99 × 10^−08^	2.17 × 10^−08^	A	0.053	__
2	rs6725478	0.104	0.018	0.123	2.13 × 10^−08^	2.28 × 10^−08^	T	0.388	UGT1A6
2	rs1105880	0.103	0.018	0.123	2.30 × 10^−08^	2.40 × 10^−08^	G	0.343	UGT1A6
2	rs1105879	0.103	0.018	0.123	2.30 × 10^−08^	2.40 × 10^−08^	C	0.325	UGT1A6
2	rs7583278	0.105	0.018	0.124	2.57 × 10^−08^	2.75 × 10^−08^	T	0.391	UGT1A6
2	rs7604115	0.107	0.019	0.122	2.57 × 10^−08^	2.76 × 10^−08^	T	0.350	UGT1A6

CHR: Chromosome. SNP: Single Nucleotide Polymorphism. Beta: indicates the regression coefficients showing the increase or decrease in the bilirubin concentrations (mg/dL) per minor allele. SE: Standard error of Beta. *R*^2^: Indicates the determination coefficient (variability explained). MA: Minor allele. MAF: minor allele (MA) frequency. *n* = 242 subjects analyzed. ^1^: Unadjusted *p*-value in the additive model. ^2^: Adjusted *p*-value for age (additive model). ^3^: SNP scored as 3a in the RegulomeDB with the meaning less likely to affect binding.

**Table 5:** GWAS results for the interaction term between the Mediterranean diet adherence and the corresponding SPN (at *p* < 1 × 10^−5^) for bilirubin concentrations in the whole sample.

		Low Adherence Mediterranean Diet ^1^		High Adherence Mediterranean Diet ^2^					
CHR	SNP	Beta1 ^3^	SE1 ^3^	Beta2 ^4^	SE2 ^4^	*p*^5^ Gene-Diet Interaction	MA	MAF	Gene
5	rs6887452	0.045	0.016	−0.087	0.018	3.14 × 10^−^^08^	G	0.397	IL17B
10	rs6585514	−0.061	0.016	0.063	0.019	5.89 × 10^−07^	A	0.395	__
6	rs7770270	−0.046	0.024	0.140	0.030	1.52 × 10^−06^	A	0.112	LAMA2
17	rs1242492	0.038	0.015	−0.074	0.018	2.78 × 10^−06^	C	0.249	__
4	rs13108021	0.052	0.017	−0.069	0.020	2.96 × 10^−06^	A	0.228	__
10	rs10886342	0.059	0.017	−0.067	0.022	4.19 × 10^−06^	A	0.277	__
18	rs12964365	−0.048	0.017	0.073	0.020	4.59 × 10^−06^	T	0.150	LOC107985179
6	rs6904763	−0.046	0.024	0.128	0.030	5.32 × 10^−06^	C	0.126	LAMA2
5	rs907195	0.054	0.017	−0.070	0.021	5.91 × 10^−06^	C	0.253	__
4	rs10305895	0.061	0.020	−0.076	0.023	6.04 × 10^−06^	G	0.341	EDNRA
1	rs11208512	0.037	0.016	−0.075	0.019	6.35 × 10^−06^	T	0.417	__
5	rs7443165	−0.046	0.019	0.089	0.024	8.62 × 10^−06^	A	0.277	__
10	rs4642993	−0.060	0.015	0.043	0.018	9.19 × 10^−06^	A	0.455	__
18	rs687862	−0.043	0.017	0.074	0.020	9.70 × 10^−06^	A	0.220	LINC01541
5	rs16871933	0.111	0.027	−0.072	0.031	9.70 × 10^−06^	G	0.081	LINC01331

CHR: Chromosome. SNP: Single Nucleotide Polymorphism. ^1^: Low Adherence to the Mediterranean Diet: from 0 to 8 points in the 17-item score (*n* = 257 subjects). ^2^: High Adherence to the Mediterranean Diet: from 9 to 17 points in the 17-item score. Beta1 ^3^: indicates the regression coefficients showing the increase or decrease in the bilirubin concentrations (mg/dL) per minor allele in the low adherence stratum. SE1 ^3^: Standard error of Beta in the low adherence stratum. Beta2 ^4^: indicates the regression coefficients showing the increase or decrease in the bilirubin concentrations (mg/dL) per minor allele in the high adherence stratum. SE2 ^4^: Standard error of Beta in the high adherence stratum. ^5^: Adjusted *p*-value for the interaction term between the Mediterranean diet adherence and the corresponding SPN. MAF: minor allele (MA) frequency. *n* = 418 subjects analyzed.

## Supplementary Materials

The following are available online at http://www.mdpi.com/2072-6643/11/1/90/s1: Table S1: Quantitative 17-item questionnaire for Adherence to Mediterranean diet. Figure S1: Graphic representation of the human UGT1A cluster, indicating the nine protein-coding (UGT1A1, UGT1A3, UGT1A4, UGT1A5, UGT1A6, UGT1A7, UGT1A8, UGT1A9, and UGT1A10) genes and the four non-coding genes (pseudogenes), as well as the process of alternative splicing with a first exon followed by a set of common exons 2–5. Table S2: Linkage disequilibrium parameters between the lead SNP (rs4148325) and the other top-ranked SNPs in chromosome 2 at the GWAS significance level for total bilirubin concentrations in the whole population. Figure S2: Q-Q plot for the GWAS on total serum bilirubin concentrations in the whole population (*n* = 430). Table S3: GWAS results (at *p* < 1 × 10^−5^) for the association between bilirubin concentrations and the top-ranked SNPs in men. Table S4: GWAS results (at *p* < 1 × 10^−5^) for the association between bilirubin concentrations and the top-ranked SNPs in women. Figure S3: Manhattan plot for the GWAS analysis on total serum bilirubin concentrations in men (A) (*n* = 188 participants) and women (B) (*n* = 242 participants) obtained in the genetic additive model adjusted for age, expressed in −log_10_(*p*-value). The blue line represents the threshold 1 (−log_10_(5 × 10^−8^)) for the GWAS statistical significance. The red line represents the threshold 2 ((−log_10_(1 × 10^−5^)). Table S5: GWAS results for the interaction term between sex and the top-ranked SNPs (at *p* < 1 × 10^−5^) for bilirubin concentrations in the whole sample. Table S6: GWAS results for the interaction term between the Mediterranean diet adherence and the corresponding SPN (at *p* < 1 × 10^−5^) for bilirubin concentrations in men. Table S7: GWAS results for the interaction term between the Mediterranean diet adherence and the corresponding SPN (at *p* < 1 × 10^−5^) for bilirubin concentrations in women. Figure S4: Q-Q plot for the GWAS on total serum bilirubin concentrations in the men (A) (*n* = 188) and women (B) (*n* = 242).
